# Biomedicine or Holistic Medicine for Treating Mentally Ill Patients? A Philosophical and Economical Analysis

**DOI:** 10.1100/tsw.2007.287

**Published:** 2007-12-18

**Authors:** Søren Ventegodt, Isack Kandel, Joav Merrick

**Affiliations:** ^1^ Quality of Life Research Center, Teglgårdstræde 4-8, DK-1452 Copenhagen K, Denmark; ^2^ Research Clinic for Holistic Medicine, Copenhagen, Denmark; ^3^ Nordic School of Holistic Medicine, Copenhagen, Denmark; ^4^ Scandinavian Foundation for Holistic Medicine, Sandvika, Norway; ^5^ Interuniversity College, Graz, Austria; ^6^ Faculty of Social Sciences, Department of Behavioral Sciences, Ariel University Center, Samaria, Ariel, Israel; ^7^ National Institute of Child Health and Human Development, Jerusalem, Israel; ^8^ Office of the Medical Director, Division for Mental Retardation, Ministry of Social Affairs, Jerusalem, Israel; ^9^ Kentucky Children's Hospital, University of Kentucky, Lexington, KY, USA

**Keywords:** clinical holistic medicine, mental health, alternative and complementary medicine, psychiatry

## Abstract

Today we have two scientific medical traditions, two schools or treatment systems: holistic medicine and biomedicine. The two traditions are based on two very different philosophical positions: subjectivistic and objectivistic. The philosopher Buber taught us that you can say I-Thou or I-It, holding the other person as a subject or an object. These two fundamentally different attitudes seem to characterize the difference in world view and patient approach in the two schools, one coming from psychoanalysis and the old, holistic tradition of Hippocratic medicine. Holistic medicine during the last decade has developed its philosophical positions and is today an independent, medical system seemingly capable of curing mentally ill patients at the cost of a few thousand Euros with no side effects and with lasting value for the patient. The problem is that very few studies have tested the effect of holistic medicine on mentally ill patients. Another problem is that the effect of holistic medicine must be documented in a way that respects this school's philosophical integrity, allowing for subjective assessment of patient benefit and using the patient as his/her own control, as placebo control cannot be used in placebo-only treatment. As the existing data are strongly in favor of using holistic medicine, which seems to be safer, more efficient, and cheaper, it is recommended that clinical holistic medicine also be used as treatment for mental illness. More research and funding is needed to develop scientific holistic medicine.

## INTRODUCTION

It is most interesting that the last century left us with two completely different, scientific traditions for treating the mentally ill: a subjectivistic and an objectivistic. These two traditions correspond exactly to the two different ways man can relate to his world, with the famous philosophical position of the existential philosopher Buber[1]: as a "thou" or as an "it". Buber published his famous book *I and Thou* in 1923 and it had quite an extreme impact on the development of the way therapists understood their own work at that time. They understood that man has only two fundamental relations in the world, either I-Thou or I-It.

Either you come from a subjectivistic position or from an objectivistic position; either you come from sheer love or from bruited force. There is no in between, no middle way, no way to mix the two. We must choose our approach and take the consequences. The person before you may be a divine being, with an autonomy equal to your own, to see, understand, and interpret the world; a center in the universe like yourself, and you have no power over this other person at all; (s)he is a unique presence, a source of existence like yourself, and you can only surrender and serve, if you want to relate at all. On the other hand is the person who regards another as an object, which can be analyzed and manipulated, trimmed, supported, or broken down; you are in power, and the other person is at your command an for your use.

The stimulus inciting Archie Cochrane's interest in scientific medicine is claimed to be his own ill health and the inability of psychoanalysis to cure his problem of sexual dysfunction[2]. The treatment undertaken by Cochrane failed because his condition was organic, and thus evidence-based medicine was founded on Cochrane's critical evaluation of his own psychoanalytic psychotherapy. The objectivistic tradition of medical science thus founded now believes that objective physical and chemical etiologies exist for all disease, not only to organic problems but also to mental problems. In psychiatry, the objectivistic approach has led to extremely complicated and detailed systems for psychiatric diagnostics related to brain-chemistry and pharmaceutical treatments thereof.

In holistic medicine, the tradition of psychodynamic therapy going back to Freud[3] and Jung[4] has been developed into a variety of ways to support patients' body, mind, spirit, and whole existence. Most popular is existentially oriented conversational therapy (see [5]), emotionally realizing bodywork[6,7,8,9], and spiritual exercises that develop the patients' consciousness and autonomy (basically taking the patients back to what Huxley called the "perennial philosophy"[10]). The holistic medical tradition believes that subjective choices and shift in consciousness is the etiology of most diseases, both somatic and mental.

### Biomedicine and Holistic Medicine in Psychiatry

In medicine, these two philosophically equally valid positions, the subjectivistic and the objectivistic, means that we as doctors either must come from an objectivistic science that tries to understand our patient by using tools for scientific analysis, diagnostics, and treatment, or from a subjectivistic science that focuses on meeting the other person soul to soul, on loving the other in an unselfish way, in letting yourself as a doctor be "the tool" in your impeccable service of the other; what you to do here is to bring the patient's subjective being into focus and surrender your own self to that meeting.

The objectivistic position is the position of the biomedical psychiatrist: the doctor has the full power to change the situation of the mentally ill patient because of his medical and scientific knowledge and competence that allows him to see and understand the mental disease as nothing more than an objective disturbance that can be treated and cured with biomedical science and its understanding of the brain and its consciousness.

The subjectivistic position is the position of the holistic doctor insisting on meeting the mentally ill patient in a nonjudging way, believing in his/her inner self-healing powers and innate knowledge and wisdom, and therefore taking the position that what is happening inside the other person is a product of this person's autonomously assumed choices and philosophical positions. As the person is obviously in trouble, the doctor surrenders his own ego and will to the situation, and coming from unselfish unconditional love, he gives what he can of attention, care, respect, acknowledgment, and acceptance to the other, in all aspects of this person's manifestations, i.e., physical, mental, spiritual, and existential.

In practice, nothing is as black and white as the two philosophical positions, but when you look for it, you can easily find these two positions as fundamental reasons for the materialization of two completely different traditions in medical science.

## Evidence-based Medicine in the Subjectivistic and Objectivistic Traditions

During the last decades, medical science crystallized its contemporary insistence on evidence-based medicine: the effect of a medical treatment must be scientifically documented. Interestingly, both the subjectivistic and the objectivistic traditions came to the acceptance of the necessity of documenting results, but naturally in the ways determined by the different nature of their approaches.

The objectivistic medical tradition has designed a genius method to exclude subjectivity, objective examinations, and pharmaceutical treatment under placebo control. Deep psychopharmacological analysis of the brain and its transmitter systems has made it possible for the doctor to interact directly with the brain's chemistry, allowing him/her to balance the brain and thereby take the person back to normal, which then can be documented by objective measurement of the symptoms of neural malfunctioning before and after, controlling for the placebo effect by giving a similar group of patients only a placebo pill. A large number of meta-analysis now exists that documents the effect of a large number of drugs on certain psychiatric symptoms in psychiatry.

The subjectivistic medical tradition has designed a similar genius method to exclude the objectivity: subjective meetings with the patient; focus on self-insight, personal development, and emotional and philosophical work; results controlled by psychometric rating of the patient's subjective evaluations of health, quality of life, and ability to function, controlled by measuring the patient's subjective state of being before and after treatment. As the intervention in holistic medicine is primarily on the patient's state of consciousness and philosophy of the world, the treatment can be understood as a treatment with placebo only, rendering the objectivistic method of placebo control completely useless and even fatal; by admitting full value to the psychometric evaluation of the patient's own subjective state of being before and after treatment (and again after 1 year to be sure of a lasting effect), the effect of therapy can be easily measured.

The most extreme and radical schools of subjectivistic therapists claim that even measuring subjectivity is objectivistic, and that evaluation of the value of treatment should be done only qualitatively, not quantitatively. As these schools might have a philosophical point, the problem is that the effect of the treatment here is going to be so "invisible" that it becomes too easy to cheat oneself as a therapist and make oneself believe that the patient has actually been helped, even when this is not the case; this problem, called the hermeneutic problem, is well known from the philosophy of science (see [11] for a review).

## Results: Which School of Medicine Helps Psychiatric Patients Best?

A search on www.pubmed.gov for "Cochrane and mental illness" gives 955 hits, "Cochrane and schizophrenia" gives 264 hits, while there are 403 hits for "Cochrane and depression". Many of these studies are meta-analyses of pharmaceutical studies.

One recent example is Adams et al. who tested chlorpromazine, the drug of choice through decades, vs. placebo for schizophrenia[12]:

> MAIN RESULTS: …We found chlorpromazine reduces relapse over the short (n=74, 2 RCTs, RR 0.29 CI 0.1 to 0.8) and medium term (n=809, 4 RCTs, RR 0.49 CI 0.4 to 0.6)

> but data are heterogeneous. Longer term homogeneous data also favoured chlorpromazine (n=512, 3 RCTs, RR 0.57 CI 0.5 to 0.7, NNT 4 CI 3 to 5). We found chlorpromazine provided a global improvement in a person's symptoms and functioning (n=1121, 13 RCTs, RR 'no change/not improved' 0.80 CI 0.8 to 0.9, NNT 6 CI 5 to 8). Fewer people allocated to chlorpromazine left trials early (n=1780, 26 RCTs, RR 0.65 CI 0.5 to 0.8, NNT 15 CI 11 to 24) compared with placebo. There are many adverse effects. Chlorpromazine is clearly sedating (n=1404, 19 RCTs, RR 2.63 CI 2.1 to 3.3, NNH 5 CI 4 to 8), it increases a person's chances of experiencing acute movement disorders (n=942, 5 RCTs, RR 3.5 CI 1.5 to 8.0, NNH 32 CI 11 to 154), parkinsonism (n=1265, 12 RCTs, RR 2.01 CI 1.5 to 2.7, NNH 14 CI 9 to 28). Chlorpromazine clearly causes a lowering of blood pressure with accompanying dizziness (n=1394, 16 RCTs, RR 2.37 CI 1.7 to 3.2, NNH 11 CI 7 to 21) and considerable weight gain (n=165, 5 RCTs, RR 4.92 CI 2.3 to 10.4, NNH 2 CI 2 to 3).

This is fine science in the objectivistic tradition; most unfortunate the results are not really good: NNT is 4 to 6, meaning that between four and six patients must be treated for one to benefit significantly. And the patients are not cured; the test is only for “improvement”. And the side effects are dramatic: weight gain in one of two, sedation to one in five, etc. The drug harms many more patients than are helped. Similar results exist for major depression; most of the meta-analysis shows NNT in the same range, although the side effects are somewhat milder; unfortunately, few studies exist of treatment of major depression vs. placebo.

The psychodynamic tradition is somewhat in between the biomedical and the holistic school, and fine meta-analysis exist, documenting that short-term psychodynamic psychotherapy (STPP) is as effective as psychiatric standard treatment in many mental disorders[14,15]. Unfortunately, STPP has not been clearly stating its “holistic” position, often using psychiatric diagnosesm etc,; the Buber “I and Thou” position is not found here much; to find it, we must continue the spectrum all the way to scientific holistic medicine, especially the recently developed “clinical holistic medicine” (CHM), which has done much to clearly state its philosophical position in the treatment of a number of diseases and illnesses[16–60].

Going to holistic medicine, a search on PubMed for “holistic medicine and meta-analysis” gave zero hits, not surprisingly. “Holistic medicine and Cochrane” gave five hits, among these a paper on the holistic strategy for testing treatment effect called the “square curve paradigm”[61], also from the CHM group, but no test results. Searching for “square curve paradigm” on www.pubmed.gov, we find two studies using this strategy for documenting effect[62,63], but and only the first study is actually testing the development of the patient’s subjective experience of mental health or illness after holistic medical treatment. The details from the results of the treatment of mental and other patients with CHM, which combines STPP, bodywork, and spiritual exercises, as holistic medicine often does, are found in several articles[64,65,66,67,68]. The treatment group in Ventegodt et al.[67] is 54 chronically ill patients that are their own control in the study; it is a diverse group of patients only having in common that they all consider themselves to be severely mentally ill before treatment.

The results are surprisingly good: 31 of 54 patients, or 57.4 (CI 43.2–70.8), were subjectively cured by the treatment consisting of 20 sessions of CHM. But as the holistic approach did not allow for specific psychiatric “objective” diagnoses, we do not know exactly how this group of patients would be diagnosed if they had gone to standard psychiatry. But what we do know is that 40% of the patients had already been to a psychiatrist, and that the chronically ill mental patient entering the clinic after abandoning psychiatry in average rated 3.7 on a 5-point Likert scale on self-evaluated mental health (from the validated questionnaire QOL5[69]), which is better than the inclusion criteria for this study: to enter, the patients needed a rating of 4 or 5 on a 5-point Likert scale, with 1 being completely mentally healthy and 5 being completely mentally ill, in their own subjective assessment. The group did not receive any drugs and only regression in the pace that their system could tolerate and did in accordance with this not report any side effects from the treatment (NNTH with CHM is estimated to 500, NNTB = 2 CI 1.4–2.3). The square curve paradigm analysis in Ventegodt et al.[62] documents that these results seems to be lasting.

The scarcity of published data from studies in peer-reviewed journals makes this study somewhat unique. Here we have a group of patients who report that they feel mentally ill before treatment, and this feeling disappears for good in half the patients after 20 sessions of CHM with no side effects. But how can we be sure that the patients in this group are really mentally ill, in the psychiatric meaning of this word? Well, we cannot. We know that 40% were treated by psychiatrist, psychologist, or GP for a mental illness, and we know that this group was helped as much as the patients that entered to the clinic directly because of their personal conviction that they needed holistic therapy. But that is all. A subjectivistic researcher would argue that what really is important is not the psychiatrist's judgment, but the patient's experience of being mentally ill or well. For it is mental pain that brings them to the doctor, and if the treatment cures this pain, without damaging other aspects of the patient, well, then the patient has been well helped.

Most interestingly, the cost of the treatment is also mentioned: 1600 Euros. Standard psychiatric treatment normally costs 10, 100, or even 1000 times this.

How can we compare the value to the patients of the treatments of these two very different medical schools, both obviously coming from fine scientific medical traditions, the biomedical and the psychoanalytic? And how do we make a fair cost/benefit analysis?

If we take the data as is from the papers, with all that uncertainty that is because one school can refer to huge meta-analyses and the other school only to single studies with few patients from the CHM group, we can see that:

1. CHM helps one in two, psychiatry helps one in five.
2. CHM cures the patient subjectively, while psychiatry takes some of the objective symptoms away, but without curing the patient.
3. CHM has no side effect, where psychiatric treatment harms more patients than it helps.
4. CHM has lasting effect, while psychiatry struggles with relapse.
5. CHM costs a few thousand Euros, while psychiatry often costs hundreds of thousands of Euros.

## DISCUSSION

We are not at all accustomed to think seriously over philosophical matters and, in this case, everything is dependent of it. As medical doctors, we are not at all used to taking a philosophical stand and we normally do not like that philosophy matters, as we are practical people who want to get things going. Deep philosophical issues should better be left to the philosophers.

However, the development of two parallel medical schools that each build on different philosophical traditions, the subjectivistic and the objectivistic, challenges this lazy attitude, for obviously our philosophical attitude matters to the treatment of our patients.

Maybe the reason why psychiatry has so little effect in helping patients is because patients are reduced to objects, and the soul, integrity, and autonomy of the patient is let down in the treatment of the patient's body, brain, and behavioral symptoms.

Most unfortunately, the methodology from objectivistic medical science is now also used to evaluate subjectivistic medical science. Cochrane tests of the value of complementary and alternative medicine (CAM) are being conducted. In the following example[70], the value of therapeutic touch is tested in anxiety disorder, therapeutic touch being common in the holistic treatment of patients[18,28]: "Inclusion criteria included all published and unpublished randomized and quasi-randomized controlled trials comparing therapeutic touch with sham (mimic) TT, pharmacological therapy, psychological treatment, other treatment or no treatment /waiting list."

The problem here is that holistic therapy does not work in either of the conditions that are listed in the list of inclusion criteria, and the insistence of placebo control, sham touch, or randomization to pharmacological therapy or psychological treatment is with absolute certainty excluding the patients that really believe in CAM, because they would never accept to be a part of a study where they could be randomized to pharmacological or psychological treatment. The only way to document therapeutic success here would be to measure the patients before and after treatment, and after a sufficiently long time, to see if their subjective assessment of own mental health really improved.

So what is happening now is that objectivistic science is evaluation subjectivistic medicine on objectivistic permission, and this is obviously not giving holistic medicine a fair trial. To test holistic medicine, the square curve paradigm or a similar method has to be used, using the patient’s own subjective evaluations of health and quality of life as endpoints, and avoiding placebo controls and other objectivistic tools that destroy the possibility of a fair scientific testing of medical interventions based on developing the patient’s consciousness.

The belief of biomedicine and psychiatry is that a placebo control is necessary to take away all disturbing subjectivity from the study. From a subjectivistic perspective, there is already a huge surrender on a philosophical and existential level when you choose psychiatric treatment, and without testing for subjective factors like patient confidence in the doctor and in this kind of medicine, the placebo effect is still 100% working on the patient and not controlled in the study. In general, the two schools have little understanding for each other’s position, and find their own arguments completely valid; only when a clear philosophical analysis is made, an openness and understanding for the other school can take place.

## CONCLUSIONS

When two equally acceptable medical systems or schools exist, as is the case with psychiatric and holistic treatment of mentally ill patients, the only rational way to choose between one or the other system is to look at the results of the treatments. But here we face a big problem, because the two schools use their own philosophic value systems as premise for the scientific evaluation. In the objectivistic medical system of psychiatry, only the treatment of objective symptoms is taken as relevant. In subjective holistic medicine, only the patient’s subjective experience of being mentally ill or well is relevant; only a subjective improvement of life at large – the state of the whole person – is relevant.

Both the holistic and the psychiatric school use valid scientific ways to treat mentally ill patients. In spite of the very limited data, it seems like to conclude that:

- Scientific holistic medicine (CHM) helps one in two, while psychiatry helps one in four or five.
- Scientific holistic medicine (CHM) cures the patient subjectively, while psychiatry takes some of the objective symptoms away, but without curing the patient.
- Scientific holistic medicine (CHM) has no side effect, where psychiatric treatment harms more patient than it helps.
- Scientific holistic medicine (CHM) has lasting effect, while psychiatry struggles with relapse.
- Scientific holistic medicine (CHM) costs a few thousand Euros while psychiatry often costs hundreds of thousands of Euros.

When it comes to treating mentally ill patients, it is recommended that holistic medicine is used; more research is necessary, and it is recommended that governments and funds support the development of scientific holistic medicine.

## References

1. Buber, M. (1970) *I and Thou*. Charles Scribner´s Sons, New York.
2. Macleod, S. (2007) Cochrane's problem: psychoanalysis and anejaculation. *Australas. Psychiatry* **15(2)**, 144–147.
3. Jones, E. (1961) *The Life and Works of Sigmund Freud*. Basic Books, New York.
4. Jung, C.G. (1964) *Man and His Symbols*. Anchor Press, New York.
5. Yalom, I.D. (1980) *Existential Psychotherapy*. Basic Books, New York.
6. Reich, W. (1969) *Die Function des Orgasmus*. Kiepenheuer & Witsch, Köln. [German]

7. Lowen, A. (2004) *Honoring the Body*. Bioenergetics Press, Alachua, FL.
8. Rosen, M. and Brenner, S. (2003) *Rosen Method Bodywork. Accessing the Unconscious Through Touch*. North Atlantic Books, Berkeley, CA.
9. Rothshild, B. (2000) *The Body Remembers*. WW Norton, New York.
10. Huxley, A. (1972) *The Perennial Philosophy*. HarperCollins, New York.
11. Chalmers, A. (1999) *What is this Thing called Science?* Open University Press, Buckingham, U.K.
12. Adams, C.E., Awad, G., Rathbone, J., and Thornley, B. (2007) Chlorpromazine versus placebo for schizophrenia. *Cochrane Database Syst. Rev*. **18(2)**, CD000284.
13. Leichsenring, F., Rabung, S., and Leibing, E. (2004) The efficacy of short-term psychodynamic psychotherapy in specific psychiatric disorders: a meta-analysis. *Arch. Gen. Psychiatry* **61(12),** 1208–1216.
14. Leichsenring, F. (2005) Are psychodynamic and psychoanalytic therapies effective? A review of empirical data. *Int. J. Psychoanal*. **86(Pt 3),** 841–868.
15. Ventegodt, S., Andersen, N.J., and Merrick, J. (2003) Holistic medicine III: the holistic process theory of healing. *TheScientificWorldJOURNAL* **3**, 1138–1146.
16. Ventegodt, S. and Merrick J. (2004) Clinical holistic medicine: applied consciousness-based medicine. *TheScientificWorldJOURNAL* **4**, 96–99.
17. Ventegodt, S., Morad, M., and Merrick, J. (2004) Clinical holistic medicine: classic art of healing or the therapeutic touch. *TheScientificWorldJOURNAL* **4**, 134–147.
18. Ventegodt, S., Morad, M., and Merrick, J. (2004) Clinical holistic medicine: the “new medicine”, the multiparadigmatic physician and the medical record. *TheScientificWorldJOURNAL* **4**, 273–285.
19. Ventegodt, S., Morad, M., and Merrick, J. (2004) Clinical holistic medicine: holistic pelvic examination and holistic treatment of infertility. *TheScientificWorldJOURNAL* **4**, 148–158.
20. Ventegodt, S., Morad, M., Hyam, E., and Merrick, J. (2004) Clinical holistic medicine: use and limitations of the biomedical paradigm *TheScientificWorldJOURNAL* **4**, 295–306.
21. Ventegodt, S., Morad, M., Kandel, I., and Merrick, J. (2004) Clinical holistic medicine: social problems disguised as illness. *TheScientificWorldJOURNAL* **4**, 286–294.
22. Ventegodt, S., Morad, M., Andersen, N.J., and Merrick, J. (2004) Clinical holistic medicine: tools for a medical science based on consciousness. *TheScientificWorldJOURNAL* **4**, 347–361.
23. Ventegodt, S., Morad, M., and Merrick, J. (2004) Clinical holistic medicine: prevention through healthy lifestyle and quality of life. *Oral Health Prev. Dent*. **1**, 239–245.
24. Ventegodt, S., Morad, M., Hyam, E., and Merrick, J. (2004) Clinical holistic medicine: when biomedicine is inadequate. *TheScientificWorldJOURNAL* **4**, 333–346.
25. Ventegodt, S., Morad, M., and Merrick, J. (2004) Clinical holistic medicine: holistic treatment of children. *TheScientificWorldJOURNAL* **4**, 581–588.
26. Ventegodt, S., Morad, M., and Merrick, J. (2004) Clinical holistic medicine: Problems in sex and living together. *TheScientificWorldJOURNAL* **4**, 562–570.
27. Ventegodt, S., Morad, M., Hyam, E., and Merrick, J. (2004) Clinical holistic medicine: holistic sexology and treatment of vulvodynia through existential therapy and acceptance through touch. *TheScientificWorldJOURNAL* **4**, 571–580.
28. Ventegodt, S., Flensborg-Madsen, T., Andersen, N.J., Morad, M., and Merrick, J. (2004) Clinical holistic medicine: a pilot stidy on HIV and quality of life and a suggested cure for HIV and AIDS. *TheScientificWorldJOURNAL* **4**, 264–272.
29. Ventegodt, S., Morad, M., and Merrick, J. (2004) Clinical holistic medicine: induction of spontaneous remission of cancer by recovery of the human character and the purpose of life (the life mission). *TheScientificWorldJOURNAL* **4**, 362–377.
30. Ventegodt, S., Morad, M., Kandel, I. and Merrick, J. (2004) Clinical holistic medicine: treatment of physical health problems without a known cause, exemplified by hypertension and tinnitus. *TheScientificWorldJOURNAL* **4**, 716–724.
31. Ventegodt, S., Morad, M., and Merrick, J. (2004) Clinical holistic medicine: developing from asthma, allergy and eczema. *TheScientificWorldJOURNAL* **4,** 936–942.
32. Ventegodt, S., Morad, M., Press, J., Merrick, J., and Shek, D.T.L. (2004) Clinical holistic medicine: holistic adolescent medicine. *TheScientificWorldJOURNAL* **4**, 551–561.
33. Ventegodt, S., Solheim, E., Saunte, M.E. Morad, M., Kandel, I., and Merrick, J. (2004) Clinical holistic medicine: metastatic cancer. *TheScientificWorldJOURNAL* **4**, 913–935.
34. Ventegodt, S., Morad, M., Kandel, I., and Merrick, J. (2004) Clinical holistic medicine: a psychological theory of dependency to improve quality of life. *TheScientificWorldJOURNAL* **4**, 638–648.
35. Ventegodt, S. and Merrick, J. (2005) Clinical holistic medicine: chronic infections and autoimmune diseases. *TheScientificWorldJOURNAL* **5**, 155–164.
36. Ventegodt, S., Kandel, I., Neikrug, S., and Merrick, J. (2005) Clinical holistic medicine: holistic treatment of rape and incest traumas. *TheScientificWorldJOURNAL* **5**, 288–297.
37. Ventegodt, S., Morad, M., and Merrick, J. (2004) Clinical holistic medicine: chronic pain in the locomotor system. *TheScientificWorldJOURNAL* **5**,165–172.

38. Ventegodt, S. and Merrick, J (2005) Clinical holistic medicine: chronic pain in internal organs. *TheScientificWorldJOURNAL* **5**, 205–210.
39. Ventegodt, S., Kandel, I., Neikrug, S., and Merrick, J. (2005) Clinical holistic medicine: the existential crisis – life crisis, stress, and burnout. *TheScientificWorldJOURNAL* **5**, 300–312.
40. Ventegodt, S., Gringols, G., and Merrick, J. (2005) Clinical holistic medicine: holistic rehabilitation. *TheScientificWorldJOURNAL* **5**, 280–287.
41. Ventegodt, S., Andersen, N.J., Neikrug, S., Kandel, I., and Merrick, J (2005) Clinical holistic medicine: mental disorders in a holistic perspective. *TheScientificWorldJOURNAL* **5**, 313–323.
42. Ventegodt, S., Andersen, N.J., Neikrug, S., Kandel, I., and Merrick, J (2005) Clinical holistic medicine: holistic treatment of mental disorders. *TheScientificWorldJOURNAL* **5**, 427–445.
43. Ventegodt, S. and Merrick, J. (2005) Clinical holistic medicine: the patient with multiple diseases. *TheScientificWorldJOURNAL* **5**, 324–339.
44. Ventegodt, S., Clausen, B., Nielsen, M.L., and Merrick, J. (2006) Clinical holistic health: Advanced tools for holistic medicine. *TheScientificWorldJOURNAL* **6**, 2048–2065.
45. Ventegodt, S., Clausen, B., and Merrick, J. (2006) Clinical holistic medicine: the case story of Anna. I. Long-term effect of childhood sexual abuse and incest with a treatment approach. *TheScientificWorldJOURNAL* **6,** 1965–1976.
46. Ventegodt, S., Calusen, B., and Merrick, J. (2006) Clinical holistic medicine: the case story of Anna. II. Patient diary as a tool in treatment. *TheScientificWorldJOURNAL* **6,** 2006–2034.
47. Ventegodt, S., Clausen, B., and Merrick, J. (2006) Clinical holistic medicine: the case story of Anna. III. Rehabilitation of philosophy of life during holistic existential therapy for childhood sexual abuse. *TheScientificWorldJOURNAL* **6,** 2080–2091.
48. Ventegodt, S. and Merrick, J. (2005) Suicide from a holistic point of view. *TheScientificWorldJOURNAL* **5**, 759–766.
49. Ventegodt, S., Clausen, B., Omar, H.A., and Merrick, J. (2006) Clinical holistic medicine: holistic sexology and acupressure through the vagina (Hippocratic pelvic massage). *TheScientificWorldJOURNAL* **6,** 2066–2079.
50. Ventegodt, S., Clausen, B., and Merrick, J. (2006) Clinical holistic medicine: pilot study on the effect of vaginal acupressure (Hippocratic pelvic massage). *TheScientificWorldJOURNAL* **6,** 2100–2116.
51. Hermansen, T.D., Ventegodt, S., Rald, E., Clausen, B., Nielsen, M.L., and Merrick, J. (2006) Human development I: twenty fundamental problems of biology, medicine, and neuro-psychology related to biological information. *TheScientificWorldJOURNAL* **6,** 747–759.
52. Ventegodt, S., Hermansen, T.D., Nielsen, M.L., Clausen, B., and Merrick, J. (2006) Human development II: we need an integrated theory for matter, life and consciousness to understand life and healing. *TheScientificWorldJOURNAL* **6**, 760–766.
53. Ventegodt, S., Hermansen, T.D., Rald, E., Flensborg-Madsen, T., Nielsen, M.L., Clausen, B., and Merrick, J. (2006) Human development III: bridging brain-mind and body-mind. introduction to "deep" (fractal, poly-ray) cosmology. *TheScientificWorldJOURNAL* **6,** 767–776.
54. Ventegodt, S., Hermansen, T.D., Flensborg-Madsen, T., Nielsen, M.L., Clausen, B., and Merrick, J. (2006) Human development IV: the living cell has information-directed self-organisation. *TheScientificWorldJOURNAL* **6,** 1132–1138.
55. Ventegodt, S., Hermansen, T.D., Flensborg-Madsen, T., Nielsen, M.L., Clausen, B., and Merrick, J. (2006) Human development V: biochemistry unable to explain the emergence of biological form (morphogenesis) and therefore a new principle as source of biological information is needed. *TheScientificWorldJOURNAL* **6,** 1359–1367.
56. Ventegodt, S., Hermansen, T.D., Flensborg-Madsen, T., Nielsen, M., and Merrick, J. (2006) Human development VI: supracellular morphogenesis. The origin of biological and cellular order. *TheScientificWorldJOURNAL* **6,** 1424–1433.
57. Ventegodt, S., Hermansen, T.D., Flensborg-Madsen, T., Rald, E., Nielsen, M.L., and Merrick, J. (2006) Human development VII: a spiral fractal model of fine structure of physical energy could explain central aspects of biological information, biological organization and biological creativity. *TheScientificWorldJOURNAL* **6,** 1434–1440.
58. Ventegodt, S., Hermansen, T.D., Flensborg-Madsen, T., Nielsen, M.L., and Merrick, J. (2006) Human development VIII: a theory of “deep” quantum chemistry and cell consciousness: quantum chemistry controls genes and biochemistry to give cells and higher organisms consciousness and complex behavior. *TheScientificWorldJOURNAL* **6,** 1441–1453.
59. Ventegodt, S., Hermansen, T.D., Flensborg-Madsen, T., Rald, E., Nielsen, M.L., and Merrick, J. (2006) Human development IX: a model of the wholeness of man, his consciousness and collective consciousness. *TheScientificWorldJOURNAL* **6,** 1454–1459.
60. Hermansen, T.D., Ventegodt, S., and Merrick, J. (2006) Human development X: Explanation of macroevolution — top-down evolution materializes consciousness. The origin of metamorphosis. *TheScientificWorldJOURNAL* **6,** 1656–1666.
61. Ventegodt, S., Andersen, N.J., and Merrick, J. (2003) The square-curve paradigm for research in alternative, complementary, and holistic medicine: a cost-effective, easy, and scientifically valid design for evidence-based medicine. *TheScientificWorldJOURNAL* **3,** 1117–1127.

62. Ventegodt, S., Thegler, S., Andreasen, T., Struve, F., Enevoldsen, L., Bassaine, L., Torp, M., and Merrick, J. (2006) Clinical holistic medicine: psychodynamic short-time therapy complemented with bodywork. A clinical follow-up study of 109 patients. *TheScientificWorldJOURNAL* **6,** 2220–2238.
63. Ventegodt, S., Clausen, B., Langhorn, M., Kroman, M., Andersen, N.J., and Merrick, J. (2004) Quality of life as medicine III. A qualitative analysis of the effect of a five-day intervention with existential holistic group therapy or a quality of life course as a modern rite of passage. *TheScientificWorldJOURNAL* **4**, 124–133.
64. Ventegodt, S., Thegler, S., Andreasen, T., Struve, F., Enevoldsen, L., Bassaine, L., Torp, M., and Merrick, J. (2007) Clinical holistic medicine (mindful, short-term psychodynamic psychotherapy complemented with bodywork) in the treatment of experienced impaired sexual functioning. *TheScientificWorldJOURNAL* **7,** 324–329.
65. Ventegodt, S., Thegler, S., Andreasen, T., Struve, F., Enevoldsen, L., Bassaine, L., Torp, M., and Merrick, J. (2007) Clinical holistic medicine (mindful, short-term psychodynamic psychotherapy complemented with bodywork) improves quality of life, health, and ability by induction of Antonovsky-salutogenesis. *TheScientificWorldJOURNAL* **7,** 317–323.
66. Ventegodt, S., Thegler, S., Andreasen, T., Struve, F., Enevoldsen, L., Bassaine, L., Torp, M., and Merrick, J. (2007). Clinical holistic medicine (mindful, short-term psychodynamic psychotherapy complemented with bodywork) in the treatment of experienced physical illness and chronic pain. *TheScientificWorldJOURNAL* **7,** 310–316.
67. Ventegodt, S., Thegler, S., Andreasen, T., Struve, F., Enevoldsen, L., Bassaine, L., Torp, M., and Merrick, J. (2007) Clinical holistic medicine (mindful, short-term psychodynamic psychotherapy complemented with bodywork) in the treatment of experienced mental illness. *TheScientificWorldJOURNAL* **7,** 306–309.
68. Ventegodt, S., Thegler, S., Andreasen, T., Struve, F., Enevoldsen, L., Bassaine, L., Torp, M., and Merrick, J. (2007). Self-reported low self-esteem. Intervention and follow-up in a clinical setting. *TheScientificWorldJOURNAL* **7,** 299–305.
69. Lindholt, J.S., Ventegodt, S., and Henneberg, E.W. (2002) Development and validation of QoL5 for clinical databases. A short, global and generic questionnaire based on an integrated theory of the quality of life. *Eur. J. Surg.* **168,** 103–107.
70. Robinson, J., Biley, F., and Dolk, H. (2007) Therapeutic touch for anxiety disorders. *Cochrane Database Syst. Rev.* **18(3),** CD006240.
